# The Arrest of Hemorrhage Following Excision of the Tonsil

**Published:** 1889-09

**Authors:** Richard J. Levis

**Affiliations:** Philadelphia


### THE ARREST OF HEMORRHAGE FOLLOWING EXCISION OF THE TONSIL.

By RICHARD J. LEVIS, M. D., OF PHILADELPHIA.

Persistent hemorrhage after excision of the tonsil is extremely rare, and I have seen but a single case in which profuse and dangerous hemorrhage continued after the operation. In that instance an unnecessary entire removal of the tonsil had been effected by the form of guillotine that draws out the mass before the cut is made.

At my first visit to the patient, a male adult, he had bled freely for some hours and was blanched and exsanguinous. Various inefficient expedients had been resorted to, including the application of cold and temporary pressure. Of course, there had been used Monsel's solution the vilest of the abominations called styptics, a class of remedies which declare the inefficiency of the surgeon, and have caused more hemorrhage by making its source and by loss of time, than they ever arrested.

After excavating from the mouth and fauces the masses of hard, black clots produced by the iron solution, the cou surface was seen bleeding freely.

All bleeding was instantly arrested by simply passing an ordinary tenaculum firmly through the tissues of the base of the tonsil, and giving the instrument a decided twist. The torsion effected, compressing the oozing vessels was maintained by closing the patient's jaws on the handle of the tenaculum as it protruded from the mouth. The jaws were then securely held together by a firm bandage about the head from the chin to the vertex.

After the arrest of hemorrhage, the patient rested through the succeeding night, experiencing apparently no annoyance from the presence of the tenaculum.

I may remark, in illustration of another expedient for arrest of hemorrhage after tonsillotomy, that at my visit on the next morning I was prepared, in case hemorrhage should recur on the removal of tenaculum, to pass one or more ligatures through the base of the tonsil, and for this purpose had ready for use needles of short curve fixed in needle-holders. A concentric constriction of the base thus made, would effectually prevent any possible hemorrhage.

In this case no hemorrhage followed on removal of the tenaculum, after it had remained twelve hours in position.—*Medical News.*

